# Imaging Posture Veils Neural Signals

**DOI:** 10.3389/fnhum.2016.00520

**Published:** 2016-10-21

**Authors:** Robert T. Thibault, Amir Raz

**Affiliations:** ^1^Integrated Program in Neuroscience, Department of Neurology and Neurosurgery, McGill UniversityMontreal, QC, Canada; ^2^The Lady Davis Institute for Medical Research at the Jewish General HospitalMontreal, QC, Canada; ^3^Department of Psychiatry, Institute for Community and Family Psychiatry, McGill UniversityMontreal, QC, Canada

**Keywords:** posture, neuroimaging, EEG, fMRI, upright, supine, cognition, perception

## Abstract

Whereas modern brain imaging often demands holding body positions incongruent with everyday life, posture governs both neural activity and cognitive performance. Humans commonly perform while upright; yet, many neuroimaging methodologies require participants to remain motionless and adhere to non-ecological comportments within a confined space. This inconsistency between ecological postures and imaging constraints undermines the transferability and generalizability of many a neuroimaging assay. Here we highlight the influence of posture on brain function and behavior. Specifically, we challenge the tacit assumption that brain processes and cognitive performance are comparable across a spectrum of positions. We provide an integrative synthesis regarding the increasingly prominent influence of imaging postures on autonomic function, mental capacity, sensory thresholds, and neural activity. Arguing that neuroimagers and cognitive scientists could benefit from considering the influence posture wields on both general functioning and brain activity, we examine existing imaging technologies and the potential of portable and versatile imaging devices (e.g., functional near infrared spectroscopy). Finally, we discuss ways that accounting for posture may help unveil the complex brain processes of everyday cognition.

## Introduction

From psychiatry and cognitive science to education and marketing, many experts draw on discoveries from human brain imaging to inform their practice. However, few consumers of neuroimaging findings fully appreciate the methodological and environmental variables that these techniques often impose. For example, in a typical functional magnetic resonance imaging (fMRI) experiment, participants lie motionless in a body-sized bore while piercing screeches, thumps, and hums thunder around their head for up to an hour. In a customary electroencephalography (EEG) experiment, participants sit upright, alone, in a small, silent, and often dimly lit room, while staring at and responding to a computer screen for extended periods of time. Of the many glaring discrepancies between such imaging environments and everyday life, this review focuses on the role of body posture. We summarize important findings from research examining the relationship between posture and brain data, highlight the mechanisms underlying these postural influences, and discuss experimental techniques that can help overcome postural caveats in human brain research.

Neuroimagers seldom draw on research suggesting that environmental variables impact human cognition. Meanwhile, an entire field of research, entitled “embodied cognition,” highlights the intricate relationship among our cognitive capacities, ongoing sensorimotor state, and surrounding environment (Thompson and Varela, 2001; Wilson, 2002; Thompson, 2005; Di Paolo and Thompson, 2014). Relevant postural findings highlight that slouching increases measures of helplessness and stress (Riskind and Gotay, 1982) and expansive postures increase testosterone, decrease cortisol, and amplify feelings of power and risk-tolerance (Carney et al., 2010). Static imaging environments further diminish cognitive loads related to balance, moving visual fields, and social interaction (Hari and Kujala, 2009). Considering these factors, some scientists demand a new neuroscientific model—the “embodied brain”—to better account for the ongoing interactions between brain, body, and environment (Kiverstein and Miller, 2015).

## Imaging methods and imaging postures

Popular functional neuroimaging modalities collect electromagnetic or hemodynamic brain data (Table 1). EEG and magnetoencephalography (MEG) record electric and magnetic signals from pyramidal neurons; fMRI measures deoxygenated blood concentrations that correlate with neural activity; and functional near infrared spectroscopy (fNIRS) measures oxygenated and deoxygenated blood flow. EEG and MEG come with spatial precision of about 1 cm, yet millisecond temporal resolution; fMRI provides millimetric spatial resolution but temporal precision of ~1 s; fNIRS excels in neither temporal nor spatial resolution and comes with a high signal-to-noise ratio compared to fMRI (Cui et al., 2011). MEG outperforms EEG in terms of signal-to-noise ratio when accessing deeper brain regions (Goldenholz et al., 2009). Each imaging modality, moreover, permits a subset of body positions. Participants can wear EEG and fNIRS caps throughout a wide range of postures (see Table 1) and, with proper equipment, can move and interact with their environment; MEG restricts participants to an adjustable seat that can adopt any position between an upright chair and a horizontal bench; and most fMRI options constrain participants to horizontal positions. Compared to portable technologies (i.e., EEG and fNIRS), the large and static imaging devices (i.e., fMRI and MEG) permit fewer posture, yet provide higher-quality data. These intrinsic differences lend certain imaging modalities more advantageous for specific applications and research questions but less so for others (e.g., the postural constraints of most MRI scanners would make fMRI a good way to explore the sleeping brain, but less ideal to study the driving brain).

**Table 1:** **Each body posture raises particular considerations in terms of brain imaging modalities and cognitive experiments**.

		**Canonical imaging postures**		**Other everyday postures**	
		**Lying supine**	**Sitting upright**	**Standing erect**	**Sitting reclined**
		** 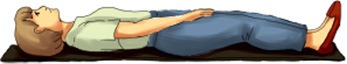 **	** 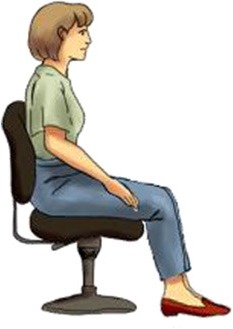 **	** 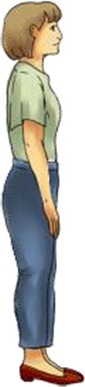 **	** 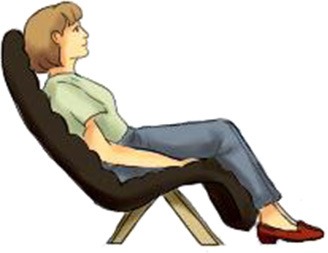 **
**EEG**	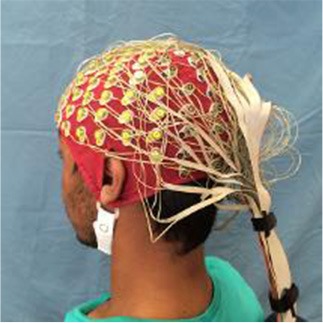	✓	✓	✓	✓
**MEG**	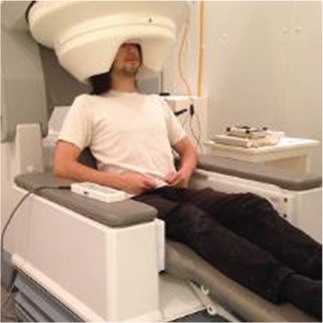	✓	✓	✘	✓
**fMRI**	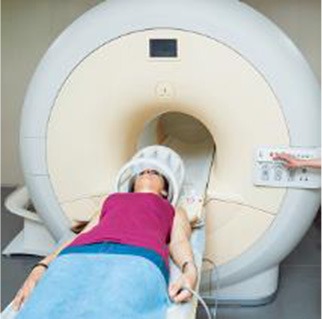	✓	✘	✘	✘
**fNIRS**	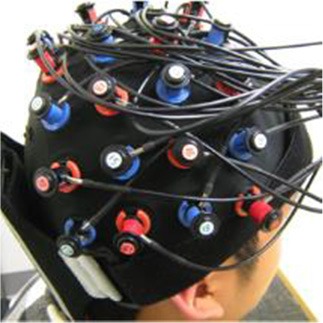	✓	✓	✓	✓
Vigilance		Low	Medium	High	Medium/low
Assumed in waking life		Rare	Common	Common	Occasional
Associated cognitive tasks		Few	Many	Many	Few
Actions possible		Few	Many	Most	Few

To conduct fMRI beyond a horizontal body posture requires specialized scanners, which are extremely uncommon. Researchers can conduct EEG and fNIRS in any posture, but must care for occipital sensors in the supine position. Humans execute most physical and cognitive actions when sitting or standing. To better depict the posture assumed in fMRI, this photo shows a participant before entering the bore. During scanning, the head and upper body remain inside the bore, which measures about 60 cm in diameter for standard scanners.

Two canonical imaging postures dominate brain research even though more ecological alternatives exist (see Table 1). These established positions include sitting upright—common in EEG, MEG, fNIRS, and most of cognitive and psychological research; and lying supine—the standard for fMRI. Whereas, a limited number of imaging experiments stray from these standardized postures, humans perform many cognitive tasks while standing and moving, yet few while lying down. Experiments leveraging non-standard body positions often ask particular questions which demand these postures. For example, researchers have participants stand or walk to better understand balance, gait, and motor disorders such as Parkinson's disease (Bakker et al., 2007; Koenraadt et al., 2014; Mahoney et al., 2016), lie supine titled 6–12° head-down past horizontal to simulate a microgravity environment (e.g., Spironelli and Angrilli, 2011), or lie prone to investigate gravitational forces on cranial fluids (Rice et al., 2013). Whereas, the execution of these experiments fully depends on the use of non-standard imaging postures, the supine and sitting positions hardly impede researchers from conducting most neuroimaging experiments. This situation may encourage neuroimagers to continue employing standardized imaging postures even when ecological comportments could better unveil the neural mechanisms of everyday cognition.

## Posture influences cognition

**Figure 1:**
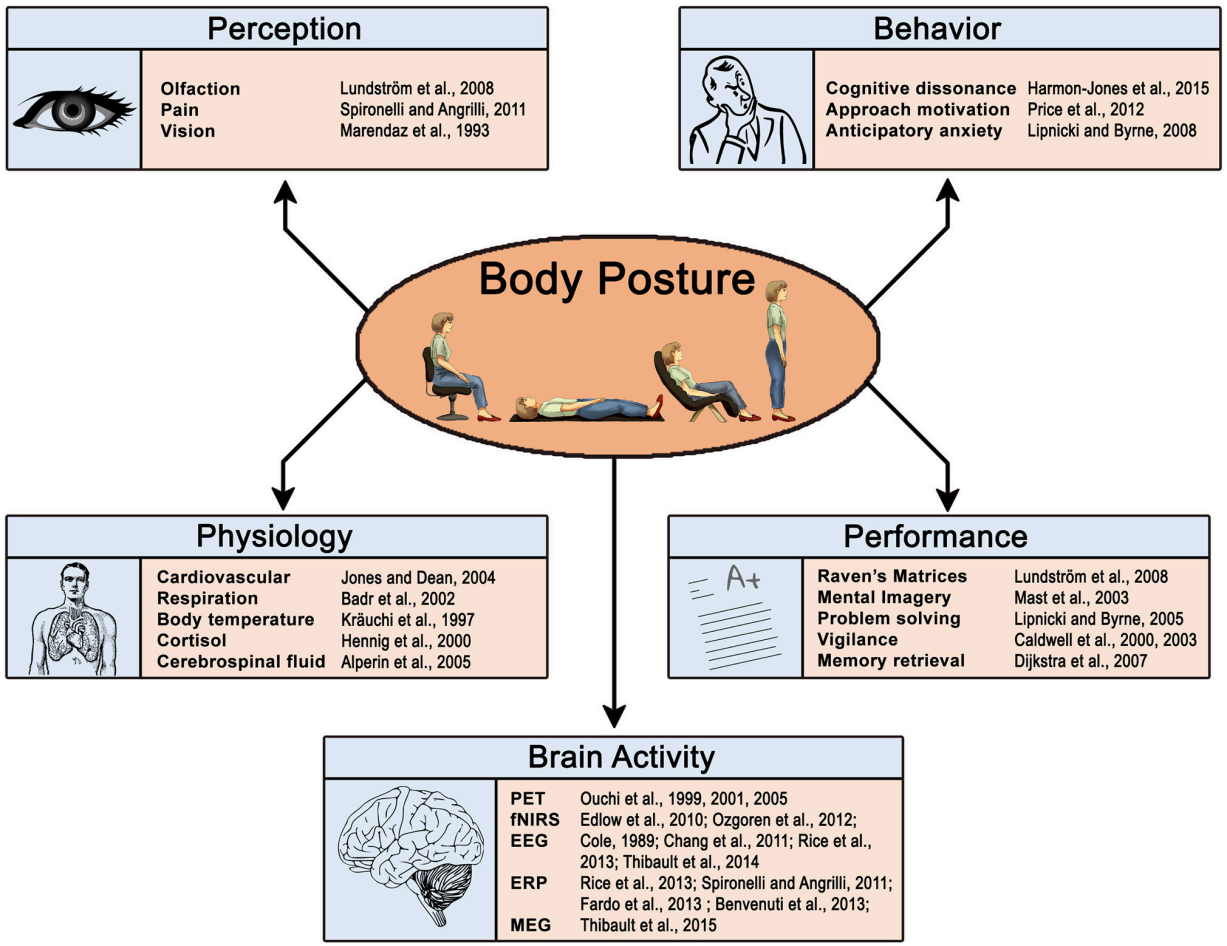
**Posture modulates physiology and cognition: Select experimental findings**.

Posture alters sensory perception and behavior (Figure 1). For example, when upright compared to supine: Olfactory thresholds increase for select odorants (e.g., Lundström et al., 2008), pain ratings amplify (e.g., Spironelli and Angrilli, 2011; Fardo et al., 2013), visual awareness improves (e.g., Goodenough et al., 1981; Marendaz et al., 1993), anticipatory anxiety heightens (e.g., Lipnicki and Byrne, 2008), approach motivation increases (Price et al., 2012), and conflicting thoughts decrease (e.g., Harmon-Jones et al., 2015). Posture further influences cognitive performance. Compared to lying supine, sitting upright improves non-verbal intelligence (e.g., Raven's Progressive Matrices; Lundström et al., 2008) and aids in composing mental images, but impairs the ability to inspect them (Mast et al., 2003). Standing compromises performance on problems requiring a burst of insight (e.g., anagrams: Lipnicki and Byrne, 2005) and improves psychomotor performance (Caldwell et al., 2000, 2003). Memories, moreover, are easier to retrieve when assuming the posture associated with the remembered event (Dijkstra et al., 2007).

The fMRI environment may alter the very phenomena researchers aim to study. This concern has motivated diverse research groups to test how posture and cognition interact (e.g., Lundström et al., 2008; Harmon-Jones and Peterson, 2009). Replication experiments, however, remain sparse, likely because posture receives more attention as a procedural caveat than a research field in its own right. Beyond posture, neuroimagers must also address several other procedural and statistical concerns before obtaining meaningful results (e.g., Eklund et al., 2016). All in all, these studies highlight the importance of considering posture across all cognitive and imaging research.

## Posture influences physiology

Heart rate, respiratory volume, oxygen consumption, core body temperature, cortisol secretion, and other indicators of physiological arousal stabilize at higher levels when upright compared to supine (Figure 1; Cole, 1989; Kräuchi et al., 1997; Hennig et al., 2000; Badr et al., 2002; Jones and Dean, 2004). These physiological differences may influence the fMRI derived blood-oxygen-level dependent (BOLD) signal, regardless of whether or not brain processes actually change (Kastrup et al., 1999; Di et al., 2013). fMRI measures neuronal activity indirectly (see Shmuel, 2015); the BOLD signal stems from the hemodynamic properties of neural populations and remains highly sensitive to cardiopulmonary variables (Chang and Glover, 2009; Chang et al., 2009; Di et al., 2013; Weinberger and Radulescu, 2016). Thus, demonstrating that posture affects the BOLD signal falls short of confirming a change in neural activity; cardiopulmonary variables remain yoked to body position and also weigh heavily on BOLD activity.

Beyond BOLD, posture governs blood flow around the brain (Gisolf et al., 2004). A few experiments employ a stance-adjustable positron emission tomography (PET) gantry and report greater blood flow to both visual and cerebellar cortices when standing erect compared to lying supine (Ouchi et al., 1999, 2001). Using fNIRS, researchers document decreases in both oxygenated and deoxygenated cortical hemoglobin volume when participants move from lying supine to sitting upright (Edlow et al., 2010; Ozgoren et al., 2012). Due to a paucity of upright MRI scanners capable of functional sequences, researchers have yet to replicate postural fNIRS experiments with fMRI. Because fNIRS and fMRI measure similar signals (Cui et al., 2011), we can only presume that postural discrepancies would also influence fMRI data.

Beyond cardiovascular measures, posture exerts a quantifiable and direct impact on neural activity. A few EEG experiments demonstrate that, compared to lying horizontally, lying head-up on an incline between 30–45° (Cole, 1989; Vaitl and Gruppe, 1992) and sitting upright (Chang et al., 2011; Spironelli et al., 2016) increase high-frequency neural activity, associated with alertness and sensory processing, and dampen down low-frequency oscillations associated with relaxed or drowsy states. More recent studies leverage high-density EEG systems and reveal greater high-frequency power across the cortex in more upright postures (Thibault et al., 2014) as well as an 80% increase in occipital gamma power when supine compared to prone (Rice et al., 2013). Posture further alters event related potentials (ERPs) in response to standard visual paradigms (Rice et al., 2013), painful stimuli (Spironelli and Angrilli, 2011; Fardo et al., 2013), and emotional processing (Price et al., 2012; Messerotti Benvenuti et al., 2013). In contrast to these findings, a recent sensor-level MEG study revealed greater high-frequency power over common language areas only, rather than the entire cortex, when sitting upright compared to when supine or reclined (Thibault et al., 2015). Sensor-level MEG results, however, may represent only the strongest postural effects and source-level analyses of such data may reveal more widespread changes reminiscent of previous EEG findings (Lifshitz et al., under review). Whereas, the majority of these studies employ healthy young adults, posture may exert a particularly strong influence on brain function in the elderly and specific patient groups (e.g., cardiovascular disease or tramautic brain injury: Ouchi et al., 2005; Thompson et al., 2005). In this regard, converging evidence from cognitive, medical, and neuroscientific research supports the “embodied brain” hypothesis and underscores the importance of postural variables in modern imaging experiments.

## Underlying mechanisms by which posture operates

At least two physiological and one cognitive mechanism contribute to the influence of posture on brain data: (1) changes in noradrenalin output, (2) altered CSF thickness, and (3) a preparatory cognitive state based on the subset of interactions possible with the environment.

1. The supine position hampers cortical excitability (Lipnicki, 2009; Spironelli et al., 2016). When lying horizontally, compared to upright, gravitational loads redistribute and stimulate arterial and cardiopulmonary baroreceptors, and in turn, lead to a reduction in sympathetic nervous system activity (Mohrman and Heller, 2003). This process appears to impede noradrenergic release from neurons in the locus coeruleus (Murase et al., 1994; Berridge and Waterhouse, 2003) and drives downstream cortical inhibition (Rau and Elbert, 2001). A cleverly designed experiment supports this theory (Cole, 1989). The researcher applied leg pressure via anti-shock trousers (normally used to treat severe blood loss) to maintain levels of baroreceptor activity between lying horizontally and lying head-up on a 40° incline. They found less high-frequency EEG activity only in the condition with reduced baroreceptor firing (i.e., 40° incline without leg pressure). Further theoretical (Lipnicki, 2009) and experimental reports (Vaitl and Gruppe, 1992; Schneider et al., 2008) support the idea that gravity initiates a physiological cascade that leads to cortical inhibition.
2. Slight shifts in CSF thickness can drastically alter EEG data (Ramon et al., 2004, 2006; Wendel et al., 2008) and, to a lesser extent, MEG data (Vorwerk et al., 2014). Strong evidence for this interaction comes from a unique two-part multi-posture MRI and EEG study (Rice et al., 2013). The researchers found that when supine compared to prone, gravity draws the brain downwards, thins out the highly conductive CSF in occipital regions by 30%, brings the brain slightly closer to posterior scalp electrodes, and in turn, amplifies high-frequency occipital EEG power by an average of 80% (Rice et al., 2013). While this study provides a wealth of information, the scarcity of erect MRI scanners likely precluded an upright condition. And yet, a complementary low-field (0.5 T) MRI study scanned participants in the seated and supine positions and found that gravity draws fluids downward into the spinal canal when upright, decreases intracranial CSF and cerebral blood flow, and amplifies intracranial compliance (Alperin et al., 2005). Measures of CSF thickness in circumscribed cortical regions, however, were not reported. Thus, the quantitative differences in CSF thickness between supine and upright postures remains largely elusive. The finding that CSF not only distorts electromagnetic brain signals, but also varies in thickness among postures, raises particular concern regarding the standard practice of using anatomical MRI data acquired in the supine posture to construct head models for EEG and MEG analyses. Whereas, postural CSF discrepancies may correlate well with brain imaging data, a clear story hardly emerges relating CSF thickness to behavioral observations. This insight suggests that factors beyond CSF likely contribute to the influence of posture on human functioning.
3. A preparatory cognitive state, set to act on the subset of possible interactions between the current position of a participant and their surrounding environment, may partially account for the influence of posture on brain activity. For example, when lying down, the brain may be poorly prepared for locomotion (de Lange et al., 2006), to observe a moving visual field (Kano, 1991), or to socially and physically interact with our environment (Hari and Kujala, 2009). Motor plans depend on ongoing limb configuration (de Lange et al., 2006), the excitability of motor cortex increases in free-standing compared to supported postures (Tokuno et al., 2009), and when sitting, compared to supine, people react more quickly to moving visual fields (Kano, 1991) and are more likely to perceive themselves as moving when exposed to a moving visual field (Guterman et al., 2012). Moreover, the supine posture decreases social behaviors (Harmon-Jones and Peterson, 2009; Price et al., 2012) and hardly invites typical social interactions known to modulate brain activity, such as eye contact (Ferri et al., 2014). These posture-dependent cognitive states may manifest in both resting-state brain oscillations (Chang et al., 2011; Thibault et al., 2014; Spironelli et al., 2016) and neural responses to stimuli (i.e., ERPs: Spironelli and Angrilli, 2011; Price et al., 2012; Fardo et al., 2013). The causality of interactions between cognition and brain activity may always remain elusive; cognitive states propel physiological change (i.e., top-down processes) and physiological parameters also weigh on cognitive states (i.e., bottom-up effects).

Taken together, physiological cascades, cranial fluids, and cognitive set all exert varying influences on brain imaging data across postures. Whereas, noradrenergic output and cognitive processing may directly influence cortical activity measured at the neuronal level, CSF shunts the transmission of electromagnetic activity from neurons to sensors and exerts little influence on neuronal activity itself. Adopting experimental designs that evaluate and integrate these three mechanisms can only help to better understand ecological human functioning.

## Correcting and accounting for the effects of posture

Two paths emerge to overcome postural caveats in neuroimaging. First, we can rework standard experimental designs to minimize the influence of posture on brain activity; and second, we can embrace new imaging technologies conducive to everyday human behavior.

Accounting for the three aforementioned postural mechanisms would require a combination of innovative experimental designs, computational expertise, and a new body of research to draw upon. For example, to maintain cortical excitability in the supine posture, researchers could entertain the possibility of applying pressure to the body via anti-shock trousers to maintain baroreceptor firing (Cole, 1989), pharmacologically sustaining noradrenalin levels, or providing periodic stimulation via conversation or sensory input to sustain participant alertness. Overcoming variation in CSF thickness may require anatomical brain scans from each participant plus compensatory algorithms to calculate the standard redistribution of CSF as a function of posture. Such algorithms do not yet exist and would demand further head modeling research that taps into a database of posture-induced CSF perturbations across individuals (e.g., see Rice et al., 2013). Novel research on posture and cognition, moreover, could help future experimental designs minimize variations in cognitive state among postures. For example, research already demonstrates that poor sleep impedes working memory when supine compared to sitting (Muehlhan et al., 2014) and hampers psychomotor performance when sitting compared to standing (Caldwell et al., 2003). These findings suggest that weeding out sleep-deprived participants from supine imaging experiments could help researchers collect brain data that better reflect upright human functioning. Neuroimagers could further benefit from extending similar screening procedures to participants with mood and hormonal disturbances in response to MRI environments (Muehlhan et al., 2011) and mental performance problems in response to scanner noise (Pripfl et al., 2006). With diligence, neuroimagers can improve current research paradigms to account for a number of these postural discrepancies.

Imaging the human brain increasingly relies on smaller, lighter, and more mobile hardware. These devices hold the potential to thrust brain imaging toward investigating everyday interactive and social cognition. With the use of overhead gantries, participants undergoing EEG and fNIRS can now move and interact in a laboratory environment (Gramann et al., 2011; Mahoney et al., 2016). Recent developments, moreover, permit individuals to connect EEG electrodes to their smartphone and record brain activity in everyday contexts (Stopczynski et al., 2014). Moving while recording EEG, however, comes with caveats. Muscle activity, eye movement, and head motion all contaminate the EEG signal, especially in high-frequency bandwidths (Muthukumaraswamy, 2013). One potential concern is that researchers who are not careful may mistake these artifacts for brain oscillations themselves. The fNIRS signal also remains sensitive to motion artifacts, but responds less to muscle contamination. These portable devices sacrifice signal quality for ecological human functioning. The use of these technologies, however, is not an “either-or” dilemma. In a single experiment, we can combine data from the more precise and static imaging modalities with data from ecological yet coarser devices. Similar to how portable devices revolutionized the field of eye-tracking (Hayhoe and Ballard, 2005), wearable neuroimaging technologies hold promise to revolutionize how we study the living human brain.

## Conclusion

Across numerous experiments, posture reliably influences brain data, core physiology, and cognitive performance. This reality rings alarm bells in a field that rarely considers postural constraints. Whereas, ecological comportments such as standing and moving recruit a host of additional brain processes and represent the base from which we perform our largest diversity of interactions, few brain imaging studies ask participants to stand or move. A pillar of neuroimaging, MRI, confines participants to a supine position seldom assumed during common wakefulness. This state of affairs brings into question the practice of using neuroimaging findings to inform our ecological behavior of everyday life. Bridging the lacuna between imaging context and ecological posture would further unveil the neural processes giving rise to the living human brain.

## Author contributions

RT reviewed the literature, consulted with experts, and prepared the initial draft. RT and AR prepared the final draft together. AR provided comments throughout manuscript preparation.

## Funding

AR acknowledges funding from the Canada Research Chair program, Discovery and Discovery Acceleration Supplement grants from the Natural Sciences and Engineering Research Council of Canada (NSERC), Canadian Institutes of Health Research, and the Bial Foundation. RT, also a Bial recipient, acknowledges an Alexander Graham Bell Canada Graduate Scholarship from NSERC. The funding sources had no involvement in reviewing the literature, writing the manuscript, or deciding to submit the paper for publication.

### Conflict of interest statement

The authors declare that the research was conducted in the absence of any commercial or financial relationships that could be construed as a potential conflict of interest.

## References

[B1] Alperin N. Hushek S. G. Lee S. H. Sivaramakrishnan A. Lichtor T. (2005). MRI study of cerebral blood flow and CSF flow dynamics in an upright posture: the effect of posture on the intracranial compliance and pressure. Acta Neurochir. Suppl. 95, 177–181. 10.1007/3-211-32318-X_38 16463846

[B2] Badr C. Elkins M. R. Ellis E. R. (2002). The effect of body position on maximal expiratory pressure and flow. Aust. J. Physiother. 48, 95–102. 10.1016/S0004-9514(14)60203-8 12047207

[B3] Bakker M. Verstappen C. C. P. Bloem B. R. Toni I. (2007). Recent advances in functional neuroimaging of gait. J. Neural Transm. 114, 1323–1331. 10.1007/s00702-007-0783-8 17622483 PMC2797840

[B4] Berridge C. W. Waterhouse B. D. (2003). The locus coeruleus-noradrenergic system: modulation of behavioral state and state-dependent cognitive processes. Brain Res. Brain Res. Rev. 42, 33–84. 10.1016/S0165-0173(03)00143-7 12668290

[B5] Caldwell J. A. Prazinko B. Caldwell J. L. (2003). Body posture affects electroencephalographic activity and psychomotor vigilance task performance in sleep-deprived subjects. Clin. Neurophysiol. 114, 23–31. 10.1016/S1388-2457(02)00283-3 12495760

[B6] Caldwell J. A. Prazinko B. F. Hall K. K. (2000). The effects of body posture on resting electroencephalographic activity in sleep-deprived subjects. Clin. Neurophysiol. 111, 464–470. 10.1016/S1388-2457(99)00289-8 10699408

[B7] Carney D. R. Cuddy A. J. C. Yap A. J. (2010). Power posing: brief nonverbal displays affect neuroendocrine levels and risk tolerance. Psychol. Sci. 21, 1363–1368. 10.1177/0956797610383437 20855902

[B8] Chang C. Cunningham J. P. Glover G. H. (2009). Influence of heart rate on the BOLD signal: the cardiac response function. Neuroimage 44, 857–869. 10.1016/j.neuroimage.2008.09.029 18951982 PMC2677820

[B9] Chang C. Glover G. H. (2009). NeuroImage relationship between respiration, end-tidal CO 2, and BOLD signals in resting-state fMRI. Neuroimage 47, 1381–1393. 10.1016/j.neuroimage.2009.04.048 19393322 PMC2721281

[B10] Chang L.-J. Lin J.-F. Lin C.-F. Wu K.-T. Wang Y.-M. Kuo C.-D. (2011). Effect of body position on bilateral EEG alterations and their relationship with autonomic nervous modulation in normal subjects. Neurosci. Lett. 490, 96–100. 10.1016/j.neulet.2010.12.034 21182897

[B11] Cole R. J. (1989). Postural baroreflex stimuli may affect EEG arousal and sleep in humans. J. Appl. Physiol. 67, 2369–2375. 2606843 10.1152/jappl.1989.67.6.2369

[B12] Cui X. Bray S. Bryant D. M. Glover G. H. Reiss A. L. (2011). A quantitative comparison of NIRS and fMRI across multiple cognitive tasks. Neuroimage 54, 2808–2821. 10.1016/j.neuroimage.2010.10.069 21047559 PMC3021967

[B13] de Lange F. P. Helmich R. C. Toni I. (2006). Posture influences motor imagery: an fMRI study. Neuroimage 33, 609–617. 10.1016/j.neuroimage.2006.07.017 16959501

[B14] Di X. Kannurpatti S. S. Rypma B. Biswal B. B. (2013). Calibrating BOLD fMRI activations with neurovascular and anatomical constraints. Cereb. Cortex 23, 255–263. 10.1093/cercor/bhs001 22345358 PMC3539449

[B15] Dijkstra K. Kaschak M. P. Zwaan R. A. (2007). Body posture facilitates retrieval of autobiographical memories. Cognition 102, 139–149. 10.1016/j.cognition.2005.12.009 16472550

[B16] Di Paolo E. A. Thompson E. (2014). The enactive approach ezequiel di paolo and evan thompson forthcoming in lawrence shapiro, Edn, in The Routledge Handbook of Embodied Cognition, ed Shapiro L. (New York, NY: Routledge Press), 68–78.

[B17] Edlow B. L. Kim M. N. Durduran T. Zhou C. Putt M. E. Yodh A. G.. (2010). The effects of healthy aging on cerebral hemodynamic responses to posture change. Physiol. Meas. 31, 477–495. 10.1088/0967-3334/31/4/002 20181999

[B18] Eklund A. Nichols T. E. Knutsson H. (2016). Cluster failure: why fMRI inferences for spatial extent have inflated false-positive rates. Proc. Natl. Acad. Sci.U.S.A. 113, 7900–7905. 10.1073/pnas.1602413113 27357684 PMC4948312

[B19] Fardo F. Spironelli C. Angrilli A. (2013). Horizontal body position reduces cortical pain-related processing: evidence from late ERPs. PLoS ONE 8:e81964. 10.1371/journal.pone.0081964 24278467 PMC3835670

[B20] Ferri F. Busiello M. Campione G. C. De Stefani E. Innocenti A. Romani G. L.. (2014). The eye contact effect in request and emblematic hand gestures. Eur. J. Neurosci. 39, 841–851. 10.1111/ejn.12428 24289090

[B21] Gisolf J. van Lieshout J. J. van Heusden K. Pott F. Stok W. J. Karemaker J. M. (2004). Human cerebral venous outflow pathway depends on posture and central venous pressure. J. Physiol. 560, 317–327. 10.1113/jphysiol.2004.070409 15284348 PMC1665206

[B22] Goldenholz D. M. Ahlfors S. P. Ha M. S. Sharon D. Ishitobi M. Vaina L. M.. (2009). Mapping the signal-to-noise-ratios of cortical sources in magnetoencephalography and electroencephalography. Hum. Brain Mapp. 30, 1077–1086. 10.1002/hbm.20571 18465745 PMC2882168

[B23] Goodenough D. R. Oltman P. K. Sigman E. Cox P. W. (1981). The rod-and-frame illusion in erect and supine observers. Percept. Psychophys. 29, 365–370. 7279560 10.3758/bf03207346

[B24] Gramann K. Gwin J. T. Ferris D. P. Oie K. Jung T. P. Lin C. T.. (2011). Cognition in action: imaging brain/body dynamics in mobile humans. Rev. Neurosci. 22, 593–608. 10.1515/RNS.2011.047 22070621

[B25] Guterman P. S. Allison R. S. Palmisano S. Zacher J. E. (2012). Influence of head orientation and viewpoint oscillation on linear vection. J. Vestib. Res. 22, 105–116. 10.3233/VES-2012-0448 23000610

[B26] Hari R. Kujala M. V. (2009). Brain basis of human social interaction : from concepts to brain imaging. Physiol. Rev. 89, 453–479. 10.1152/physrev.00041.2007 19342612

[B27] Harmon-Jones E. Peterson C. K. (2009). Supine body position reduces neural response to anger evocation. Psychol. Sci. 20, 1209–1210. 10.1111/j.1467-9280.2009.02416.x 19656336

[B28] Harmon-Jones E. Price T. F. Harmon-Jones C. (2015). Supine body posture decreases rationalizations: testing the action-based model of dissonance. J. Exp. Soc. Psychol. 56, 228–234. 10.1016/j.jesp.2014.10.007

[B29] Hayhoe M. Ballard D. (2005). Eye movements in natural behavior. Trends Cogn. Sci. 9, 188–194. 10.1016/j.tics.2005.02.009 15808501

[B30] Hennig J. Friebe J. Ryl I. Krämer B. Böttcher J. Netter P. (2000). Upright posture influences salivary cortisol. Psychoneuroendocrinology 25, 69–83. 10.1016/S0306-4530(99)00037-2 10633536

[B31] Jones A. Dean E. (2004). Body position change and its effect on hemodynamic and metabolic status. Heart Lung 33, 281–290. 10.1016/j.hrtlng.2004.04.004 15454907

[B32] Kano C. (1991). An ecological theory of motion sickness and postural instability an ecological theory of motion sickness and postural instability. Ecol. Psychol. 3, 241–252. 10.1207/s15326969eco0303

[B33] Kastrup A. Krüger G. Glover G. H. Moseley M. E. (1999). Assessment of cerebral oxidative metabolism with breath holding and fMRI. Magn. Reson. Med. 42, 608–611. 10.1002/(SICI)1522-2594(199909)42:3<608::AID-MRM26>3.0.CO;2-I 10467308

[B34] Kiverstein J. Miller M. (2015). The embodied brain: towards a radical embodied cognitive neuroscience. Front. Hum. Neurosci. 9:237. 10.3389/fnhum.2015.00237 25999836 PMC4422034

[B35] Koenraadt K. L. M. Roelofsen E. G. J. Duysens J. Keijsers N. L. W. (2014). Cortical control of normal gait and precision stepping: an fNIRS study. Neuroimage 85, 415–422. 10.1016/j.neuroimage.2013.04.070 23631980

[B36] Kräuchi K. Cajochen C. Wirz-Justice A. (1997). A relationship between heat loss and sleepiness : effects of postural change and melatonin administration. J. Appl. Physiol. 83, 134–139. 9216955 10.1152/jappl.1997.83.1.134

[B37] Lipnicki D. M. (2009). Baroreceptor activity potentially facilitates cortical inhibition in zero gravity. Neuroimage 46, 10–11. 10.1016/j.neuroimage.2009.01.039 19457387

[B38] Lipnicki D. M. Byrne D. G. (2005). Thinking on your back: solving anagrams faster when supine than when standing. Brain Res. Cogn. Brain Res. 24, 719–722. 10.1016/j.cogbrainres.2005.03.003 16099373

[B39] Lipnicki D. M. Byrne D. G. (2008). An effect of posture on anticipatory anxiety. Int. J. Neurosci. 118, 227–237. 10.1080/00207450701750463 18205079

[B40] Lundström J. N. Boyle J. A. Jones-Gotman M. (2008). Body position-dependent shift in odor percept present only for perithreshold odors. Chem. Senses 33, 23–33. 10.1093/chemse/bjm059 17761723

[B41] Mahoney J. R. Holtzer R. Izzetoglu M. Zemon V. Verghese J. Allali G. (2016). The role of prefrontal cortex during postural control in Parkinsonian syndromes a functional near-infrared spectroscopy study. Brain Res. 1633, 126–138. 10.1016/j.brainres.2015.10.053 26551767 PMC4860007

[B42] Marendaz C. Stivalet P. Barraclough L. Walkowiac P. (1993). Effect of gravitational cues on visual search for orientation. J. Exp. Psychol. Hum. Percept. Perform. 19, 1266–1277. 10.1037/0096-1523.19.6.1266 8294891

[B43] Mast F. W. Ganis G. Christie S. Kosslyn S. M. (2003). Four types of visual mental imagery processing in upright and tilted observers. Cogn. Brain Res. 17, 238–247. 10.1016/S0926-6410(03)00111-3 12880895

[B44] Messerotti Benvenuti S. Bianchin M. Angrilli A. (2013). Posture affects emotional responses: a head down bed rest and ERP study. Brain Cogn. 82, 313–318. 10.1016/j.bandc.2013.05.006 23792473

[B45] Mohrman D. E. Heller L. J. (2003). Cardiovascular Physiology. New York, NY: Lange Medical Books/McGraw-Hill.

[B46] Muehlhan M. Lueken U. Wittchen H. U. Kirschbaum C. (2011). The scanner as a stressor: evidence from subjective and neuroendocrine stress parameters in the time course of a functional magnetic resonance imaging session. Int. J. Psychophysiol. 79, 118–126. 10.1016/j.ijpsycho.2010.09.009 20875462

[B47] Muehlhan M. Marxen M. Landsiedel J. Malberg H. Zaunseder S. (2014). The effect of body posture on cognitive performance: a question of sleep quality. Front. Hum. Neurosci. 8:171. 10.3389/fnhum.2014.00171 24723874 PMC3973903

[B48] Murase S. Inui K. Nosaka S. (1994). Baroreceptor inhibition of the locus coeruleus noradrenergic neurons. Neuroscience 61, 635–643. 10.1016/0306-4522(94)90440-5 7969934

[B49] Muthukumaraswamy S. D. (2013). High-frequency brain activity and muscle artifacts in MEG/EEG: a review and recommendations. Front. Hum. Neurosci. 7:138. 10.3389/fnhum.2013.00138 23596409 PMC3625857

[B50] Ouchi Y. Okada H. Yoshikawa E. Futatsubashi M. Nobezawa S. (2001). Absolute changes in regional cerebral blood flow in association with upright posture in humans: an orthostatic PET study. J. Nucl. Med. 42, 707–712. 11337564

[B51] Ouchi Y. Okada H. Yoshikawa E. Nobezawa S. Futatsubashi M. (1999). Brain activation during maintenance of standing postures in humans. Brain 122, 329–338. 10071060 10.1093/brain/122.2.329

[B52] Ouchi Y. Yoshikawa E. Kanno T. Futatsubashi M. Sekine Y. Okada H.. (2005). Orthostatic posture affects brain hemodynamics and metabolism in cerebrovascular disease patients with and without coronary artery disease: a positron emission tomography study. Neuroimage 24, 70–81. 10.1016/j.neuroimage.2004.07.044 15588598

[B53] Ozgoren M. Tetik M. Izzetoglu K. Oniz A. Onaral B. (2012). Effect of body position on NIRS based hemodynamic measures from prefrontal cortex, in Lecture Notes in Computer Science, eds Liu D. Alippi C. Zhao D. Hussain A. (Berlin; Heidelberg: Springer), 138–146.

[B54] Price T. F. Dieckman L. W. Harmon-Jones E. (2012). Embodying approach motivation: body posture influences startle eyeblink and event-related potential responses to appetitive stimuli. Biol. Psychol. 90, 211–217. 10.1016/j.biopsycho.2012.04.001 22522185

[B55] Pripfl J. Robinson S. Leodolter U. Moser E. Bauer H. (2006). EEG reveals the effect of fMRI scanner noise on noise-sensitive subjects. Neuroimage 31, 332–341. 10.1016/j.neuroimage.2005.11.031 16414278

[B56] Ramon C. Schimpf P. Haueisen J. Holmes M. Ishimaru A. (2004). Role of soft bone, CSF and gray matter in EEG simulations. Brain Topogr. 16, 245–248. 10.1023/B:BRAT.0000032859.68959.76 15379221

[B57] Ramon C. Schimpf P. H. Haueisen J. (2006). Influence of head models on EEG simulations and inverse source localizations. Biomed. Eng. Online 5:10. 10.1186/1475-925X-5-10 16466570 PMC1389789

[B58] Rau H. Elbert T. (2001). Psychophysiology of arterial baroreceptors and the etiology of hypertension. Biol. Psychol. 57, 179–201. 10.1016/S0301-0511(01)00094-1 11454439

[B59] Rice J. K. Rorden C. Little J. S. Parra L. C. (2013). Subject position affects EEG magnitudes. Neuroimage 64, 476–484. 10.1016/j.neuroimage.2012.09.041 23006805

[B60] Riskind J. H. Gotay C. C. (1982). Physical posture: could it have regulatory or feedback effects on motivation and emotion? Motiv. Emot. 6, 273–298. 10.1007/BF0099224927065904

[B61] Schneider S. Brümmer V. Carnahan H. Dubrowski A. Askew C. D. Strüder H. K. (2008). What happens to the brain in weightlessness? A first approach by EEG tomography. Neuroimage 42, 1316–1323. 10.1016/j.neuroimage.2008.06.010 18606233

[B62] Shmuel A. (2015). Locally measured neuronal correlates of functional MRI signals, in fMRI: From Nuclear Spins to Brain Functions, eds Uludağ K. Uğurbil K. Berliner L. (New York, NY: Springer), 63–82.

[B63] Spironelli C. Angrilli A. (2011). Influence of body position on cortical pain-related somatosensory processing: an ERP study. PLoS ONE 6:e24932. 10.1371/journal.pone.0024932 21949794 PMC3174221

[B64] Spironelli C. Busenello J. Angrilli A. (2016). Supine posture inhibits cortical activity: evidence from Delta and Alpha EEG bands. Neuropsychologia 89, 125–131. 10.1016/j.neuropsychologia.2016.06.015 27312745

[B65] Stopczynski A. Stahlhut C. Larsen J. E. Petersen M. K. Hansen L. K. (2014). The smartphone brain scanner: a portable real-time neuroimaging system. PLoS ONE 9:e86733. 10.1371/journal.pone.0086733 24505263 PMC3914802

[B66] Thibault R. T. Lifshitz M. Jones J. M. Raz A. (2014). Posture alters human resting-state. Cortex 58, 199–205. 10.1016/j.cortex.2014.06.014 25041937

[B67] Thibault R. T. Lifshitz M. Raz A. (2015). Body position alters human resting-state: insights from multi-postural magnetoencephalography. Brain Imaging Behav. 10, 772–780. 10.1007/s11682-015-9447-8 26409469

[B68] Thompson E. (2005). Sensorimotor subjectivity and the enactive approach to experience. Phenomenol. Cogn. Sci. 4, 407–427. 10.1007/s11097-005-9003-x

[B69] Thompson E. Varela F. (2001). Radical embodiment: Neural dynamics and conscious experience. Trends Cogn. Sci. 5, 418–425. 10.1016/S1364-6613(00)01750-2 11707380

[B70] Thompson J. Sebastianelli W. Slobounov S. (2005). EEG and postural correlates of mild traumatic brain injury in athletes. Neurosci. Lett. 377, 158–163. 10.1016/j.neulet.2004.11.090 15755518

[B71] Tokuno C. D. Taube W. Cresswell A. G. (2009). An enhanced level of motor cortical excitability during the control of human standing. Acta Physiol. 195, 385–395. 10.1111/j.1748-1716.2008.01898.x 18774948

[B72] Vaitl D. Gruppe H. (1992). Body position and changes in EEG. J. Psychophysiol. 6, 111–118.

[B73] Vorwerk J. Cho J.-H. Rampp S. Hamer H. Knösche T. R. Wolters C. H. (2014). A guideline for head volume conductor modeling in EEG and MEG. Neuroimage 100, 590–607. 10.1016/j.neuroimage.2014.06.040 24971512

[B74] Weinberger D. R. Radulescu E. (2016). Finding the elusive psychiatric “lesion” with 21st-Century neuroanatomy: a note of caution. Am. J. Psychiatry. 173, 27–33. 10.1176/appi.ajp.2015.15060753 26315983

[B75] Wendel K. Narra N. G. Hannula M. Kauppinen P. Malmivuo J. (2008). The influence of CSF on EEG sensitivity distributions of multilayered head models. IEEE Trans. Biomed. Eng. 55, 1454–1456. 10.1109/TBME.2007.912427 18390339

[B76] Wilson M. (2002). Six views of embodied cognition. Psychon. Bull. Rev. 9, 625–636. 10.3758/BF03196322 12613670

