# Corrigendum: Imaging Posture Veils Neural Signals

**DOI:** 10.3389/fnhum.2017.00045

**Published:** 2017-02-01

**Authors:** Robert T. Thibault, Amir Raz

**Affiliations:** ^1^Integrated Program in Neuroscience, Department of Neurology and Neurosurgery, McGill UniversityMontreal, QC, Canada; ^2^The Lady Davis Institute for Medical Research at the Jewish General HospitalMontreal, QC, Canada; ^3^Department of Psychiatry, Institute for Community and Family Psychiatry, McGill UniversityMontreal, QC, Canada

**Keywords:** posture, neuroimaging, EEG, fMRI, upright, supine, cognition, perception

Reason for Corrigendum:

In the original article, there was an error. A correction has been made to the Introduction, section ‘Imaging Methods and Imaging Postures’, first paragraph.

The sentence “MEG outperforms EEG in terms of signal-to-noise ratio when accessing deeper brain regions (Goldenholz et al., 2009)” should read “MEG outperforms EEG in terms of signal-to-noise ratio when accessing **superficial** brain regions (Goldenholz et al., 2009).”

The authors apologize for this error and state that this does not change the scientific conclusions of the article in any way.

## Conflict of interest statement

The authors declare that the research was conducted in the absence of any commercial or financial relationships that could be construed as a potential conflict of interest.
